# The Relationship Between Hepcidin-Mediated Iron Dysmetabolism and COVID-19 Severity: A Meta-Analysis

**DOI:** 10.3389/fpubh.2022.881412

**Published:** 2022-04-26

**Authors:** Denggao Peng, Yanzhang Gao, Li Zhang, Zhichao Liu, Huan Wang, Yingxia Liu

**Affiliations:** ^1^Shenzhen Third People's Hospital, Second Hospital Affiliated to Southern University of Science and Technology, Shenzhen, China; ^2^Graduate Collaborative Training Base of Shenzhen Third People's Hospital, Hengyang Medical School, University of South China, Hengyang, China

**Keywords:** COVID-19, ferritin, hepcidin, iron metabolism, severity

## Abstract

**Backgrounds:**

Hepcidin has been identified as a systemic iron-regulatory hormone. Recent studies have suggested that iron metabolism disorders may be involved in the pathogenesis of acute respiratory distress syndrome and multiple organ dysfunction in coronavirus disease 2019 (COVID-19).

**Objectives:**

To re-evaluate the hepcidin-related iron metabolism parameters and explore the relationship between hepcidin-mediated iron dysmetabolism and COVID-19 severity.

**Methods:**

COVID-19 is classified as mild and moderate as non-severe, severe and critical as severe. A meta-analysis was conducted. Four bibliographic databases were comprehensively searched up to December 31st 2021.

**Results:**

Six unique studies with data from 477 COVID-19 patients were included. Compared to non-severe cases, severe cases had higher hepcidin (standardized mean difference (SMD), −0.39; 95% Confidence Interval (CI) [−0.76, −0.03]; *P* = 0.03) and ferritin (SMD, −0.84; 95% CI [−1.30, −0.38]; *P* = 0.0004). In five out of six studies, a total of 427 patients were tested for serum iron, and there were significant differences in their levels between severe and non-severe cases (SMD, 0.22; 95% CI [0.02, 0.41]; *P* = 0.03). A total of 320 patients from four out of six studies were tested for transferrin saturation, and the statistical difference was not significant (SMD, 0.06; 95% CI [−0.17, 0.28]; *P* = 0.64).

**Conclusion:**

Severe COVID-19 cases had higher serum levels of hepcidin and ferritin, and lower serum iron, without significant differences in transferrin saturation. Further studies are needed to verify whether targeting the hepcidin-mediated iron metabolism axis may influence the outcome and treatment of COVID-19.

## Introduction

Since the outbreak of coronavirus disease 2019 (COVID-19) caused by severe acute respiratory syndrome coronavirus 2 (SARS-CoV-2) (1), the number of confirmed cases has increased rapidly around the world (2, 3). Acute respiratory distress syndrome (ARDS) and multiple organ dysfunction are the main clinical manifestations and leading causes of mortality in severe COVID-19 cases (4). Iron homeostasis is crucial for host immune defense and inflammatory response. Disorders of iron metabolism are mainly manifested as iron deficiency and/or overload, both of which can cause cellular and organ dysfunction (5). Low serum iron (SI) concentration restricts hemoglobin synthesis and causes anemia and systemic hypoxemia. Emerging data have found that hypoferremia is an independent risk factor for hypoxic respiratory failure and death in COVID-19 patients (6, 7). The rapid and excessive accumulation of intracellular iron, especially in macrophages, causes cell and tissue damage, presumably through the production of reactive oxygen species (ROS) catalyzed by iron. In addition, iron overload may also trigger a unique iron-dependent form of non-apoptotic cell death termed ferroptosis (8). Serum ferritin concentration has been proven to reflect the individual's iron storage status. Studies have found that increased serum ferritin levels are associated with adverse outcomes (9–11). Although studies on transferrin saturation (TSAT) have been far fewer than that of ferritin and hypoferritemia, low TSAT has been consistently reported in severe COVID-19, and significantly correlated to lung aeration loss, inflammatory markers and worse outcomes (7, 12–14).

The hepatic peptide hepcidin has been identified as the systemic iron-regulatory hormone and plays an important role in maintaining iron homeostasis (15). Hepcidin regulates intestinal iron absorption, SI concentrations, and tissue iron redistribution by inducing the degradation of its receptor, the sole iron exporter ferroportin (14, 16), and blocking ferroportin export activity (17). Serum hepcidin measurement may be a promising tool for assessing the status of iron metabolism (18), but has not been widely implemented in clinical practice (14, 19, 20). Notably, unlike serum ferritin testing that has been documented in guidelines, hepcidin has been rarely measured in COVID-19 patients (13, 21). As expected in severe inflammatory diseases, recent studies have reported that most COVID-19 patients showed varying degrees of upregulation of hepcidin levels (16, 21–25). More importantly, hepcidin levels have significantly negatively correlated with the ratio of arterial oxygen partial pressure (PaO_2_) and fraction of inspired oxygen (FiO_2_), which can be used to predict COVID-19 severity and mortality (22). To adopt the results for clinical use, these interesting findings need to be validated in wider cohorts, and the relationship between hepcidin-mediated iron dysmetabolism and COVID-19 severity also needs to be further analyzed. In view of the particularity of the prevention and control of the COVID-19 epidemic and the randomness of its incidence, large-scale, multi-center, prospective cohort studies are very difficult to achieve. Here we classify mild and moderate as non-severe, severe, and critical as severe, and conduct a meta-analysis to re-evaluate the hepcidin-related iron metabolism parameters (hepcidin, ferritin, iron, and TSAT) in COVID-19 patients and explore the relationship between iron dysmetabolism and severity.

## Methods

### Research Strategy

This meta-analysis was performed following a recently published guideline and reported according to PRISMA (Preferred Reporting Items for Systematic Reviews and Meta-Analyses) guidelines (26). We searched comprehensively MEDLINE (National Library of Medicine, US), EMBASE (Elsevier, Netherlands), Cochrane (Cochrane Collaboration, UK), and the WHO COVID-19 database to identify relevant articles up to December 31, 2021. Keywords include “COVID-19,” “SARS-CoV-2,” and iron metabolism biomarkers, including “hepcidin,” “ferritin,” “serum iron,” “transferrin,” “soluble transferrin receptor,” “unsaturated iron binding capacity,” and “transferrin saturation,” The detailed search strategy is given in Supplementary File 1.

### Study Selection and Eligibility Criteria

We follow the international guidelines for the diagnosis and treatment of COVID-19. The detailed severity classification criteria used in the evaluation of original studies and meta-analyses include: (1) Mild: asymptomatic or mild clinical symptoms, and no pneumonia on imaging, or outward treatment; (2) Moderate: fever, respiratory tract symptoms, pneumonia on imaging, and inward treatment; (3) Severe: dyspnea, or respiratory rate >30 breaths/min at rest, or oxygen saturation <93%, or PaO_2_/FiO_2_ < 300 mmHg; (4) Critical: respiratory failure and need for mechanical ventilation, or shock, or combined with other organ failure should be treated in the Intensive Care Unit. Severe and critical categories were defined as severe, mild, and moderate as non-severe in data analysis.

Inclusion criteria:

(1) All observational studies (e.g., cross-sectional, longitudinal, case-control, and cohort) with prospective or retrospective designs.
(2) Studies published in English, including preprints.
(3) Studies investigating hepcidin-mediated iron metabolism in COVID-19 cases.

Exclusion criteria:

(1) Duplicate studies, or repeated published studies.
(2) Studies in which accurate data cannot be obtained or some data are missing.
(3) Studies not strictly grouped by severity.

### Data Extraction

The extraction methodology was discussed and formulated by the team. Two of us (ZL and HW) independently screened the full text according to the selection criteria, and recorded information about the author's name, publication time, study location, study design, sample size, laboratory results of iron metabolism parameters, and COVID-19 severity in the data file. All laboratory values were converted to conventional units based on the US National Institute of Standards and Technology conversion factors. For studies that only reported the median and interquartile range, we converted these values into mean and standard deviation according to the methods reported by Hozo et al. (27) and Wan et al. (28).

### Risk of Bias Assessment

Two authors (LZ and YG) independently used the Newcastle-Ottawa scale to assess the quality of the included studies (29). A third author (DP) ruled in case consensus was not reached. The scale was developed for non-random and observational studies including case-control, cross-sectional, longitudinal, and cohort studies (if applicable). The quality was assessed using a 9-star system. Studies rated ≥6 stars were defined as “acceptable quality” and proceeded to the final meta-analysis step.

### Statistical Analysis

This meta-analysis used RveMan5.4 (Cochrane Collaboration), with inverse variance as the statistical method to calculate standardized mean differences (SMDs) and corresponding 95% confidence intervals (CIs) for continuous variables. A *P* < 0.05 was determined to be statistically significant. The *I*^2^ statistic was used to assess the heterogeneity among the analyzed studies. An *I*^2^ > 50% or *P* < 0.10 indicated heterogeneity, for which the random effects model was used. Otherwise, the fixed effects model was used. In addition, visual inspection of funnel plots was used to evaluate publication bias.

## Results

### Study Identification and Selection

**Figure 1:**
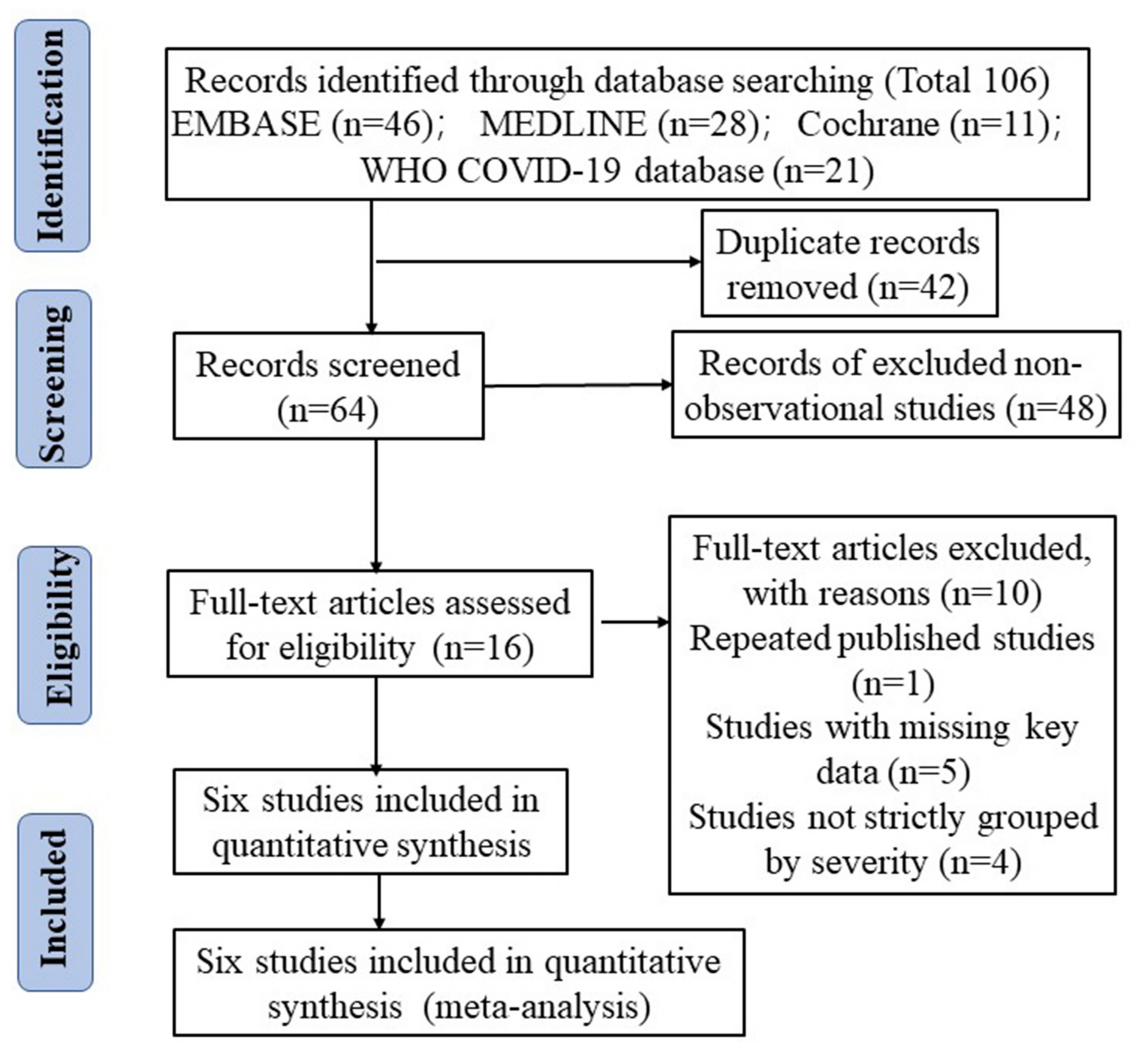
The literature selection process based on the PRISMA flow diagram.

Sixty-four unique citations were identified, of which 48 non-observational studies were excluded and 16 were selected for full-text eligibility assessment. Of these, six observational studies including 477 COVID-19 patients were included in the final data analysis and synthesis. The flow chart and details of study selection results can be found in Figure 1 and Supplementary File 1. The detailed characteristics of the included studies were shown in Table 1.

**Table 1:** Detailed characteristics of the eligible studies.

**References**	**Region**	**Center**	**Design**	**Sample size**	**Cohort settings**	**Iron parameter**	**NOS score**
Chakurkar et al. (24)	India	Single	Prospective, cohort, longitudinal	120	Mild (*n* = 22); Moderate (*n* = 57); Severe (*n* = 41)	Hepcidin, Ferritin, SI, TSAT	7*
Duca et al. (25)	Italy (Piacenza)	Single	Prospective, cohort, longitudinal	32	PaO_2_/FiO_2_ ratio: >300 mmHg (*n* = 13) >200, <300 (*n* = 14) <100 (*n* = 5)	Hepcidin, Ferritin, SI, TSAT	6*
Nai et al. (22)	Italy (Milano)	Single	Retrospective, cohort, cross -sectional	107	PaO_2_/FiO_2_ ratio: >300 (*n* = 50) <300 (*n* = 57)	Hepcidin, Ferritin, SI	7*
Sonnweber et al. (16)	Austria	Multiple	Prospective, cohort, cross -sectional	109	Mild (*n* = 22); Moderate (*n* = 34); Severe (*n* = 53)	Hepcidin, Ferritin, SI, TSAT	8*
Yagci et al. (23)	Turkey	Single	Retrospective, cohort, cross -sectional	59	Mild (*n* = 18); Severe (*n* = 19); Critical (*n* = 22)	Hepcidin, Ferritin, SI, TSAT	7*
Zhou et al. (21)	China	Single	Retrospective, cohort, cross -sectional	50	Mild (*n* = 38); Severe (*n* = 12)	Hepcidin, Ferritin	6*

FiO_2_, fraction of inspired oxygen; NOS, Newcastle-Ottawa quality assessment scale; PaO_2_, arterial oxygen partial pressure; SI, serum iron; TSAT, transferrin saturation. The symbol * means stars.

### Serum Levels of Iron Metabolism Parameters

**Figure 2:**
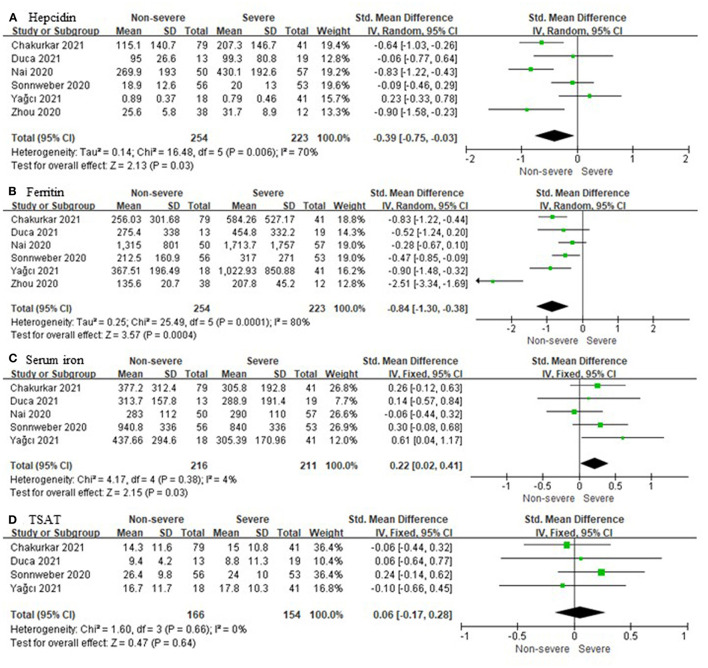
Comparison of iron metabolism parameters between non-severe and severe COVID-19 cases. **(A)** Hepcidin; **(B)** Ferritin; **(C)** Serum iron; **(D)** Transferrin saturation (TSAT).

Six unique studies with data from 477 COVID-19 patients were included. Hepcidin and ferritin were assessed in six out of six (100%) studies. Heterogeneity was assessed at *P* = 0.006, *I*^2^ = 70% and *P* = 0.0001, *I*^2^ = 80%, respectively (Figures 2A,B). Both of them had significant heterogeneity, and the random effects model was used. Compared to non-severe cases, severe cases have higher hepcidin (SMD, −0.39; 95% CI [−0.76, −0.03]; *P* = 0.03) and ferritin (SMD, −0.84; 95% CI [−1.30, −0.38]; *P* = 0.0004). In five out of six (83.3%) studies, a total of 427 patients were tested for SI, and there were significant differences in their levels between severe and non-severe cases (SMD, 0.22; 95% CI [0.02, 0.41]; *P* = 0.03). A total of 320 patients from four out of six (66.7%) studies were tested for TSAT, and the statistical difference was insignificant (SMD, 0.06; 95% CI [−0.17, 0.28]; *P* = 0.64). Heterogeneity was assessed at *P* = 0.38, *I*^2^ = 4% and *P* = 0.66, *I*^2^ = 0%, respectively (Figures 2C,D). Neither of them was significantly heterogenous, and the fixed effects model was used.

### Quality of Studies and Publication Bias

All six included studies had NOS scores ≥6 stars, and their quality was acceptable. The funnel plots of SMDs in hepcidin and ferritin (both included six studies) were asymmetric, suggesting the possible presence of publication bias. For SI and TAST, five and four studies were included, respectively, and we found no evidence of publication bias (Supplementary File 1).

## Discussion

To our knowledge, this study is the first meta-analysis of the relationship between hepcidin-mediated iron dysmetabolism and COVID-19 severity. However, there is significant heterogeneity among six studies included in the data synthesis of hepcidin and ferritin. The possible reasons are analyzed as follows: (a) The time point and time course of laboratory testing were different. Some studies collected blood samples at admission (21–23), another 2 months after onset (16), and others throughout the hospital stay (24, 25). In addition, the detection methods and the reagents used were not the same. Enzyme-linked immunosorbent assay (ELISA) kits (16, 21–24) or competitive enzyme immunoassay (EIA) kits (25) were used to detect hepcidin. (b) Iron metabolism is strongly influenced by gender, with females generally having lower SI and hepcidin levels than males (22). Also, males have a higher ratio of severe COVID-19 cases. There were potential effects on heterogeneity due to differences in the composition of males and females between studies. (c) Our study classified mild and moderate as non-severe, severe and critical as severe. Differences in the proportions and sample sizes of cases of distinct severity between studies contributed to heterogeneity. Unfortunately, hepcidin and its related iron metabolism parameters are rarely tested simultaneously in COVID-19, and there are even fewer original studies analyzing the relationship between hepcidin and disease severity. Also, subgroup analysis could not be performed due to the lack of access to raw data. Furthermore, only the Sonnweber 2020 study tested hepcidin and ferritin 2 months after onset. We removed this study and performed a new meta-analysis and found no significant change in the final overall effect (Supplementary Figure 1). Therefore, from the results of the meta-analysis, differences in blood collection at different stages of COVID-19 between studies may not affect the fact that severe cases have higher levels of hepcidin and ferritin. This also suggests that hepcidin-mediated iron dysmetabolism throughout the course of COVID-19 may be some very promising therapeutic targets. Comprehensively, these studies met rigid criteria for inclusion and were homogeneous in terms of study design and objectives, as well as cohort settings. Therefore, the final overall effect generated by the random effects model brings novel insights into the biological effects of COVID-19.

Hepcidin, the key iron metabolism regulatory hormone, sequesters iron and prevents iron efflux in enterocytes and macrophages, resulting in increased intracellular ferritin and hypoferremia (30, 31). Physiologically, hepcidin synthesis by hepatocytes is reactively upregulated or downregulated by high or low SI, respectively. Other hepcidin agonists are inflammatory cytokines represented by interleukin-6 (IL-6). Conversely, hepcidin is antagonized by hypoxemia, with hypoxia induced factors (HIF) release (15, 32). Our study found that severe COVID-19 cases had higher serum levels of hepcidin and ferritin, and lower SI, without significant differences in TSAT.

These findings were consistent with most of other studies. Unexpectedly, hepcidin levels in severe COVID-19 cases did not appear to be downregulated by hypoferremia in a feedback manner and antagonized by systemic hypoxemia. Hyperferritinemia inevitably promotes the production of ROS and lipoperoxidation, which ultimately leads to extensive cell and tissue damage possibly through the ferroptosis pathway, and cascade-amplified inflammation (32, 33). In turn, hyperinflammation will transcriptionally upregulate hepcidin, further exacerbating iron dysmetabolism. We speculate that the potential intervention targets for this vicious circle may lie in the hepcidin-mediated iron metabolism axis.

Cytokine storm is a hallmark of the hyperinflammatory state of COVID-19 and is closely linked to disturbances in iron metabolism (30, 34). A growing body of studies have suggested that several components of elevated inflammatory status may be effective therapeutic targets, notably the administration of IL-6 receptor antagonists such as tocilizumab, which significantly reduces the mortality of severe COVID-19 cases (35, 36). However, the effective suppression of the excessive inflammatory response has also raised concerns about prolonged SARS-CoV-2 clearance. Furthermore, data on whether IL-6 receptor antagonists can correct hepcidin-mediated iron dysmetabolism in COVID-19 cases have been lacking to date.

Ehsani highlighted the striking similarity between the amino acid sequence of the SARS-CoV-2 spike glycoprotein and the hepcidin protein (37). This observation provides ideas for vaccine design and bioengineered antibody development for SARS-CoV-2. Hepcidin-mimetic action of SARS-CoV-2 may markedly increase circulating and tissue ferritin, while inducing SI deficiency (32, 33). However, whether SARS-CoV-2 utilizes ferroportin on the cell membrane as another binding receptor for the spike protein to invade host cells requires further analysis. Moreover, whether SARS-CoV-2 directly degrades ferroportin through mimicking hepcidin or indirectly upregulates hepcidin through inflammation to mediate iron dysmetabolism also needs to be carefully investigated. In animal models, hepcidin-neutralizing monoclonal antibodies have been shown to reverse inflammatory anemia (38). Recently, an antibody targeting ferroportin has been described and hypothesized to reduce ferroportin degradation by interfering with hepcidin binding, thereby increasing SI (39). The application of hepcidin antibodies to block viral entry and correct iron metabolism disturbances in COVID-19 cases may be a very promising therapeutic approach in the future.

Hyperferritinemia in COVID-19 lies downstream of the hepcidin-mediated iron metabolism axis. Iron chelation has been shown to reduce viral replication and exert anti-inflammatory effects in viral infections (32, 40, 41). Multiple evidences have shown that deferoxamine reduces the levels of IL-6, the main inflammatory mediator that triggers cytokine storm, mimics HIF, and downregulates hepcidin (40, 42). However, the application of iron chelators in COVID-19 has brought great controversy (42–44). The iron dysmetabolism of COVID-19 is essentially abnormal iron distribution; the coexistence of low SI and local intracellular iron overload, and total iron in the body may not increase. Therefore, the safety and efficacy of systemic iron chelator administration has been challenged (43), and hepcidin antagonists may be preferred as supportive treatments for COVID-19 compared with iron chelators. Four clinical trials of iron chelation are currently underway (NCT04333550, NCT04361032, NCT04389801, IRCT20200506047323N4) and the results are pending.

There are some limitations to this study that need to be noted. First, most of the studies were single-center, retrospective, with small sample sizes, and may be subject to confounding and bias. Second, the six studies used for hepcidin and ferritin data synthesis were heterogeneous, subgroup analyses were not performed, and the results from random-effects models may be inaccurate. Third, the literature search may not be completely comprehensive, resulting in the omission of a few relevant studies. Fourth, this included preprint is a preliminary manuscript version. There are certain risks to the validity and applicability of the data it provides.

## Conclusions

Severe COVID-19 cases had higher serum levels of hepcidin and ferritin, and lower SI, without significant differences in TSAT. Compared with other clinically applied therapeutic options targeting the iron metabolism axis, such as IL-6 receptor antagonists and iron chelators, hepcidin antibody may be more promising, but further studies are needed to verify whether targeting the hepcidin-mediated iron metabolism axis may influence the outcome and treatment of COVID-19.

## Data Availability Statement

The original contributions presented in the study are included in the article/Supplementary Material, further inquiries can be directed to the corresponding author/s.

## Author Contributions

DP was responsible for methodology, investigation, formal analysis, data curation, writing the original draft, and visualization. YG and LZ for investigation, formal analysis, and data curation. YL for conceptualization, investigation, review and editing, and supervision. ZL and HW worked on conceptualization, formal analysis, investigation, and data curation. All authors have read and approved the final manuscript version to be submitted.

## Conflict of Interest

The authors declare that the research was conducted in the absence of any commercial or financial relationships that could be construed as a potential conflict of interest.

## Publisher's Note

All claims expressed in this article are solely those of the authors and do not necessarily represent those of their affiliated organizations, or those of the publisher, the editors and the reviewers. Any product that may be evaluated in this article, or claim that may be made by its manufacturer, is not guaranteed or endorsed by the publisher.

## Abbreviations

ARDS: acute respiratory distress syndrome
CI: confidence interval
COVID-19: coronavirus disease 2019
FiO_2_: fraction of inspired oxygen
HIF: hypoxia induced factor
NOS: Newcastle-Ottawa quality assessment scale
PaO_2_: arterial oxygen partial pressure
SARS-CoV-2: severe acute respiratory syndrome coronavirus 2
SMD: standardized mean difference
SI: serum iron
TSAT: transferrin saturation.
