# Supplementary figures and images for: The Relationship Between Hepcidin-Mediated Iron Dysmetabolism and COVID-19 Severity: A Meta-Analysis
Source: Front Public Health. 2022 Apr 26;10:881412. doi: 10.3389/fpubh.2022.881412 (PMC9087037; doi:10.3389/fpubh.2022.881412)

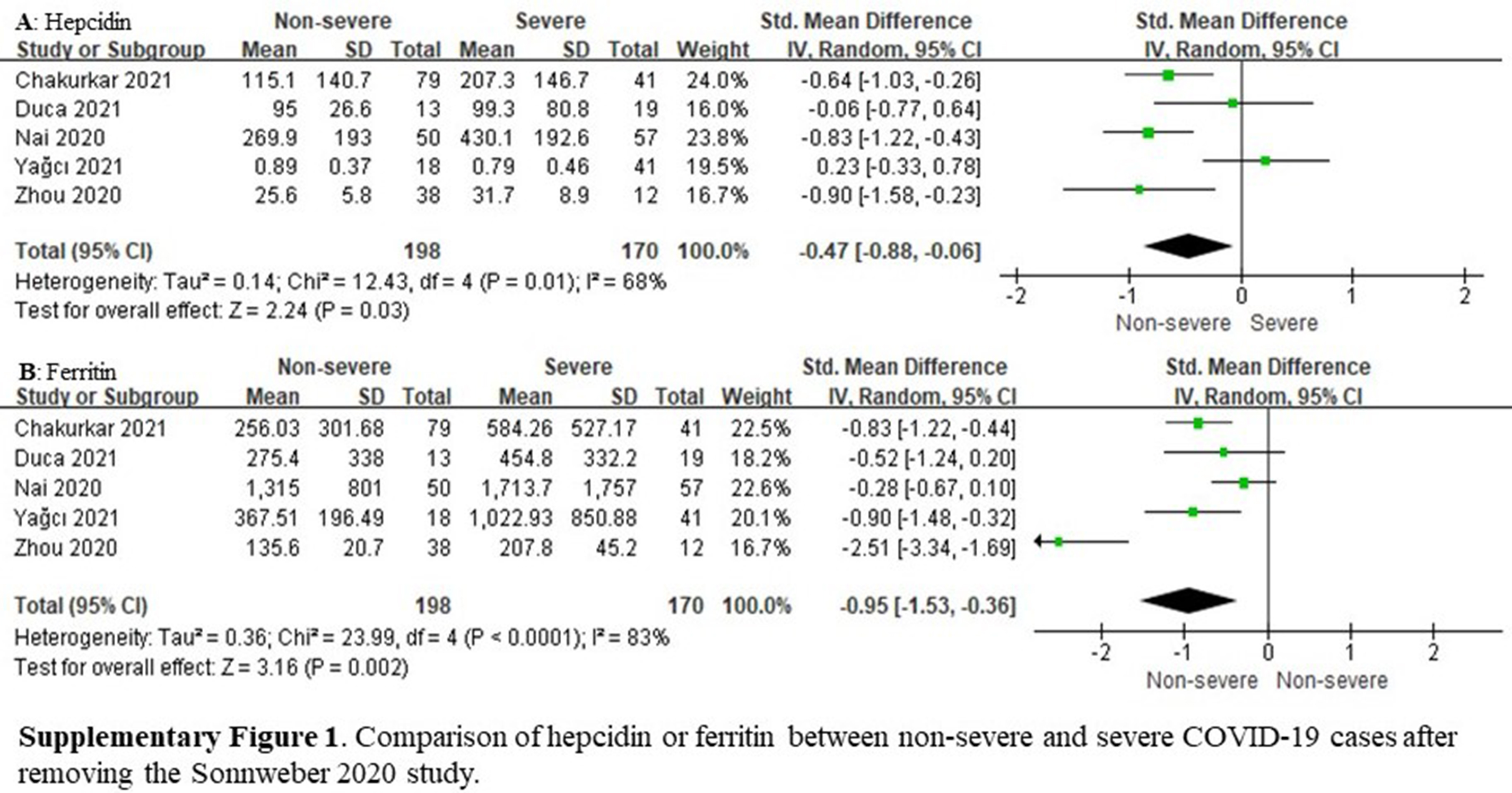

Supplement: Supplementary file 1 [file Image_1.JPEG]
